# The antifungal activity and mechanism of action of limonene against tobacco-isolated *Fusarium oxysporum* causing tobacco root rot

**DOI:** 10.3389/fpls.2026.1897693

**Published:** 2026-08-12

**Authors:** Wenjuan Yang, Wenhui Yin, Hui Liu, Yong Yang, Yao Qin, Yanyan Li, Haibo Xiang

**Affiliations:** 1 Hubei Provincial Key Laboratory of Occurrence and Intervention of Kidney Diseases, Hubei Provincial Engineering Research Center of Immunotherapy Drugs for Renal Tumors, School of Medicine, Hubei Polytechnic University, Huangshi, China; 2 State Key Laboratory of Biocatalysis and Enzyme Engineering, College of Life Sciences, Hubei University, Wuhan, China; 3 Hubei Provincial Tobacco Research Institute, Hubei Provincial Key Laboratory of Germplasm Innovation and Quality Regulation for Special Economic Crops, Wuhan, China

**Keywords:** antifungal mechanism, *Fusarium oxysporum*, limonene, multi-omics, tobacco root rot

## Abstract

Tobacco root rot (TRR), caused by *Fusarium oxysporum*, poses a significant threat to tobacco production. Synthetic fungicides widely used to control this soil-borne pathogen frequently cause fungicide resistance and environmental contamination. Limonene, a natural terpene with potent antifungal activity, features low residue and high biosafety, rendering it a promising natural substitute for chemical fungicides against TRR. This study aimed to investigate the antifungal activity and mechanisms of limonene against *F. oxysporum* using *in vitro* assays, physiological tests, transcriptomics, and metabolomics. The results demonstrated that limonene significantly inhibited mycelial growth and spore proliferation, with an EC_50_ of 201.2 μg/mL (1.48 mM). It exhibited a dose-dependent repellent effect on the chemotactic behavior of *F. oxysporum*, with effective concentrations as low as 25 μg/mL (0.18 mM). Furthermore, it disrupted cell membrane integrity and permeability at concentrations as low as 100 μg/mL (0.73 mM). Transcriptomic screening identified 3,871 differentially expressed genes (DEGs), which were significantly enriched in ribosome, proteasome, ER protein processing, autophagy and glyoxylate metabolism pathways. Eight representative key DEGs (*cat1*, *rhogap*, *tf*, *cyc*, *bro1*, *mic12*, *rpl13*, *clptm1l*) related to fungal growth and stress response were verified by RT-qPCR verification, and their expression trends were consistent with the transcriptomic results. Metabolomic analysis revealed 626 differential accumulated metabolites (DAMs), three key metabolites (ortho-Hydroxyphenylacetic acid, 4-Methylphenol and Lenticin) were significantly altered under limonene treatment. These DAMs were mainly enriched in starch and sucrose, riboflavin, folate and tyrosine metabolism pathways. Multi-omics joint analysis indicated that key targeted pathways including energy metabolism, amino acid metabolism, lipid metabolism, and protein synthesis and degradation were regulated. In conclusion, limonene inhibits *F. oxysporum* through multiple mechanisms, including cell membrane damage, chemotaxis blockage, transcriptional regulation, and metabolic disturbance. This study provides scientific support for the development of limonene as a green botanical fungicide for controlling tobacco root rot.

## 1 Introduction

Tobacco (*Nicotiana tabacum* L.) is an important cash crop widely cultivated in more than 100 countries and regions worldwide (Zhang et al., 2025). In recent years, the outbreak and spread of *Fusarium* root rot have posed a serious threat to tobacco production. This disease has affected approximately 10% to 20% of tobacco fields in several major tobacco-producing provinces in China and has gradually become one of the most destructive soil-borne fungal diseases of tobacco (Gai et al., 2021; Qiu et al., 2022; Shen et al., 2023). The pathogens responsible for this disease are primarily fungi of the genus *Fusarium*, with *Fusarium oxysporum* being the most frequently reported major pathogenic species (Gai et al., 2022; Liu et al., 2024; Pandey, 2023). As a cosmopolitan soil-borne plant pathogenic fungus, *F. oxysporum* can also infect various crops, including vegetables, flowers, field crops, and economically important forest fruits, leading to significant economic losses (Michielse and Rep, 2009). Currently, synthetic fungicides serve as the standard benchmark to evaluate antifungal activity against *F. oxysporum*, yet excessive application induces pathogen resistance, environmental residues and human health threats as reported previously (Álvarez-Martínez et al., 2020). Therefore, developing green and efficient prevention and control technologies is crucial for the sustainable development of tobacco leaf production.

Natural products, primarily derived from the secondary metabolites of plants, animals, bacteria, and fungi, are valued for their diverse sources, environmental friendliness, enhanced safety towards non-target organisms, varied mechanisms of action, and reduced tendency to induce resistance. These products have found extensive applications in pharmaceuticals, health products, and the chemical industry. Furthermore, they are considered an ideal alternative or supplementary tool to chemical pesticides for controlling plant diseases and pests (Álvarez-Martínez et al., 2020; Porras et al., 2021). Limonene is one such representative natural product with potent antifungal activity.

Limonene is a monocyclic monoterpene compound that is widely present in plants such as citrus, mint, eucalyptus, and pines (Lin et al., 2024). It exhibits significant anti-inflammatory, antioxidant, and antitumor activities. In agriculture, limonene demonstrates multiple biological effects, including insecticidal, fungicidal, and herbicidal properties (Marei et al., 2012). Furthermore, it can act as an adjuvant to enhance pesticide efficacy, showing promising potential for application in the control of agricultural pests, diseases, and weeds. Current studies indicate that limonene can inhibit the plant pathogen *F. oxysporum*, as well as other *Fusarium* species, including *F. graminearum*, *F. proliferatum*, and *F. verticillioides* (Jian et al., 2023; Krishnakumar et al., 2025). A large body of prior literature has documented limonene’s inhibitory activity toward fungal and bacterial pathogens from cereals, fruits, and vegetables (Cheng et al., 2022; Ibáñez et al., 2020; Kim et al., 2024; Víchová et al., 2024). Related research also shows that essential oils rich in limonene can suppress horticultural strains of *F. oxysporum* (Abdel-Kawy et al., 2021; Muñoz Castellanos et al., 2020; Mondal et al., 2026). However, few systematic analyses have focused on the molecular mechanisms through which pure limonene restrains tobacco-originated *F. oxysporum*. On this basis, this study systematically investigated the inhibitory effect of pure limonene on tobacco-isolated *F. oxysporum* and preliminarily elucidated its potential molecular regulatory patterns. This work hopes to enrich fundamental insights into botanical pesticide action mechanisms and provide auxiliary theoretical and technical support for the green prevention of tobacco *Fusarium* root rot.

## 2 Materials and methods

### 2.1 Materials and reagents

The pathogenic *Fusarium oxysporum* strain, previously isolated from symptomatic tobacco, was preserved in our laboratory and cultured on PDA medium (200 g/L potato peels, 20 g/L dextrose, 15 g/L agar, pH 7.0) (Almjalawi et al., 2023). Limonene and propidium iodide (PI) were purchased from Shanghai Macklin Biochemical Technology Co., Ltd.

### 2.2 Antifungal effect of limonene against *F. oxysporum*

The inhibitory activity of limonene on the mycelial growth of *F. oxysporum* was determined with reference to the method of Morcia et al. (2017). A limonene stock solution was prepared using ethanol as the solvent. Mycelial discs (5 mm) from the edge of a 7-day-old *F. oxysporum* plate were placed in the center of fresh PDA plates containing different concentrations of limonene (10, 20, 50, 100, 200, 500 μg/mL, corresponding to 0.07, 0.15, 0.37, 0.73, 1.47, 3.67 mM). PDA plates containing only 0.5% (v/v) ethanol were used as solvent controls. After cultured at 28 °C for 7 d, the diameters of the mycelial discs were recorded, and the mycelial growth inhibition rate (IR%) was calculated as follows: Inhibition rate (%) = (Diameter of control group − Diameter of limonene treatment group)/Diameter of control group × 100%. The logarithm of limonene concentration was plotted against the corresponding inhibition rate, and nonlinear regression fitting (four-parameter logistic model) was performed using GraphPad Prism 10.1.2 to calculate the half-maximal effective concentration (EC_50_).

### 2.3 Determination of limonene’s inhibitory effect on *F. oxysporum* by microplate method

Following the methodology established by Makambi et al. (2023), a 200 μL reaction system was constructed using a 96-well cell culture plate to evaluate the inhibitory effect of limonene on the growth of *F. oxysporum*. The system contained 100 μL of *F. oxysporum* spore suspension (1×10^5^ spores/mL), different volumes of limonene solution, and the remainder was supplemented with sterile PDB medium to make the final mass concentrations of limonene 12.5, 25.0, 50.0, 100.0, 200.0, 500.0 μg/mL. Control groups (0.5% v/v ethanol) and background blank groups (only PDB medium) were set up, with 3 biological replicates in each group. After sterile sealing, the culture plate was incubated at 28 °C in the dark, and samples were taken at 0, 2, 4, and 6 days. The OD_600_ value was measured with a microplate reader to quantitatively evaluate the antifungal effect.

### 2.4 Chemotaxis assay

The chemotaxis test of *F. oxysporum* was carried out with reference to the method of David et al. (2015). *F. oxysporum* spores were washed with sterile water, filtered through a 70 μm sterile filter, and the concentration was adjusted to 2.5 × 10^6^ spores/mL, sterilized 0.7% water agar was cooled to 40 °C, mixed with the spore suspension, and 20 mL was poured into a sterile petri dish. After solidification, it was blown dry for 20 min. Parallel grooves with a width of 1 mm were made 5 mm on both sides of the midline of the plate, and 50 μL of limonene solutions with different concentrations (12.5, 25, 50, 100, 200, 400 μg/mL, equivalent to 0.09, 0.18, 0.37, 0.73, 1.47, 2.94 mM) were added to each groove. Meanwhile, negative controls (equal volume of ethanol with the same concentration) and positive controls (10 mmol/L glucose solution) were set up. The plate was sealed and cultured at 28 °C in the dark for 24–36 h, and mycelial growth was terminated at 4 °C. The number of spore-germinated mycelia facing the grooves in the same area on both sides of the midline was observed under a bright-field microscope with a 20× objective lens. A total of 100 mycelia were randomly counted per plate, with 3 biological replicates for each concentration.

### 2.5 Propidium iodide staining of *F. oxysporum* mycelia

Propidium Iodide (PI) staining was used to determine the cell membrane integrity of *F. oxysporum* with the method described by Ke et al. (2023). Briefly, PDB liquid media containing 100 μg/mL (0.73 mM), 200 μg/mL (1.47 mM), and 400 μg/mL (2.94 mM) limonene were prepared, with the ethanol serving as control. *F. oxysporum* mycelial discs (5 mm) cultured at 28 °C for 7 days were inoculated into each medium and cultured with shaking at 28 °C and 180 r/min for 12 h. Following this, the cultures were centrifuged at 8000 r/min at 4 °C for 5 min to collect mycelia, which were washed 3 times with PBS buffer and resuspended in 1.5 mL of PBS buffer. PI was added to the samples to a final concentration of 10 μg/mL, stained with shaking at room temperature in the dark for 20 min, followed by three washes with PBS to remove unbound dyes. Observation was performed with a fluorescence microscope (excitation wavelength 535 nm, emission wavelength 617 nm). Three fields of view were randomly selected for each group, and the experiment was independently repeated 3 times.

### 2.6 Determination of cellular content leakage

Time gradients of 6, 12, and 24 h were set to monitor the damage effect of limonene on the cell membrane of *F. oxysporum*. The relative conductivity of the extracellular solution was measured as described by Chen and Cooper (2002), and the concentration of extracellular soluble protein was determined by the Bradford (1976). In brief, mycelial discs (5 mm) from the edge of *F. oxysporum* colonies cultured at 28 °C for 7 days were inoculated into 100 mL of PDB medium and cultured with shaking at 28 °C and 180 r/min for 3 days. Mycelia were harvested, washed 3 times with PBS, and 1 g of fresh mycelia were incubated in sterile water containing 100, 200, or 400 μg/mL (0.73, 1.47, or 2.94 mM) limonene, with ethanol serving as control (CK). At 6, 12, and 24 h of culture, the supernatant was collected by centrifugation to measure relative electrical conductivity and soluble protein concentration. All treatments were set with 3 biological replicates.

### 2.7 Transcriptome sequencing and analysis

Mycelial discs (5 mm) from the edge of *F. oxysporum* colonies cultured at 28 °C for 7 days were transferred to PDB liquid medium containing 200 μg/mL (1.47 mM) limonene, and a blank control group without limonene was set up, with 3 biological replicates in each group. After culturing at 28 °C and 180 r/min for 24 h, the mycelia were collected, and total RNA was extracted using the Fungal Total RNA Isolation Kit (Sangon Biotech, China). RNA purity and concentration were assessed via agarose gel electrophoresis and a NanoDrop 2000 spectrophotometer (Thermo Scientific, USA). Sequencing libraries were constructed with the NEBNext^®^ Ultra™ II Directional RNA Library Prep Kit (Santacruz et al., 2022) (NEB, USA) and sequenced on an Illumina NovaSeq 6000 platform with a paired-end 150 bp (PE150) strategy by Novogene Co., Ltd.

The raw data obtained from sequencing were filtered using FastQC and Trimmomatic software to obtain clean data, requiring Q20 ≥ 90% and Q30 ≥ 85%; the clean data were aligned with the *F. oxysporum* reference genome, and gene read counts were counted using the R language HTSeq R package and converted to FPKM values for quantification. Differentially expressed genes (DEGs) were screened using the DESeq2 R package with Padjust < 0.05 and |log_2_FC| > 1 as criteria. Sample reproducibility was evaluated by PCA, and volcano plots and heatmaps were drawn to visualize the expression patterns of DEGs. The clusterProfiler R package was used in combination with the GO database (https://geneontology.org/) and KEGG database (https://www.genome.jp/kegg/) to screen significantly enriched GO terms and KEGG pathways with *P* < 0.05, and all analyses were set with three technical replicates.

### 2.8 Untargeted metabolomics and data analysis

A total of 500 mg of mycelia treated above were resuspended in 5 mL of methanol-acetonitrile-water mixture (2:2:1, v/v/v), added with 20 μL of 1 mg/mL L-2-chlorophenylalanine methanol internal standard solution, vortexed for 3 min, and then sonicated at 4 °C and 200 W for 30 min (3 s sonication, 2 s interval); the samples were incubated at -20 °C for 2 h, centrifuged at 13000 r/min at 4 °C for 15 min, and the supernatant was filtered through a 0.22 μm organic phase filter membrane. A total of 2 μL of the filtrate was injected for UHPLC-MS/MS analysis, which was performed by BGI Genomics Co., Ltd. UHPLC was performed on an Agilent 1290 system equipped with a Waters ACQUITY UPLC HSS T3 column (100 mm×2.1 mm, 1.8 μm) at a flow rate of 0.3 mL/min with gradient elution: 5% B maintained for 1 min, 5%-95% B eluted for 9 min, 95% B maintained for 10 min, 95%-5% B switched at 10.1 min, and 5% B maintained for 12 min (mobile phase A was aqueous solution containing 0.1% formic acid or 5 mM ammonium acetate solution (pH 9.0), and mobile phase B was acetonitrile); MS was performed on an AB SCIEX Triple Quad™ 6500+ triple quadrupole mass spectrometer with electrospray ionization (ESI) in positive/negative ion modes, scanning range of 50-1000 (m/z), spray voltage of 5500 V for positive ions and -4500 V for negative ions, ion source temperature of 550 °C, and qualitative and quantitative analysis using MRM combined with IDA mode.

Raw UHPLC-MS/MS data were processed with Analyst 1.6.3 software for peak alignment, extraction, and metabolite quantification. The peak area was calibrated with the first QC sample, with a mass deviation tolerance of 5 ppm and a retention time deviation tolerance of 0.2 min; peak intensity was normalized by internal standard, and metabolites were matched and qualitatively/quantitatively analyzed by combining HMDB and Metlin databases. Statistical analysis was performed using R software, and compounds with CV > 15% in QC samples were eliminated. Metabolites were annotated through KEGG (https://www.genome.jp/kegg/), HMDB (https://hmdb.ca/), and LIPID Maps databases (http://www.lipidmaps.org/). The validity of the model was verified by PLS-DA combined with permutation test on metaX software. Differential metabolites were screened with fold change ≥ 2 or ≤ 0.5, *P* < 0.05, and VIP > 1 as criteria. Volcano plots were generated using the ggplot2 package, classification of identified metabolites was implemented, and KEGG pathway enrichment analysis was performed with the clusterProfiler package (Fisher’s exact test, P < 0.05).

### 2.9 Quantitative real-time PCR validation

The total RNA of *F. oxysporum* was extracted and qualified with the method above. The PrimeScript™ RT Reagent Kit (TaKaRa, Japan) was used for reverse transcription to synthesize the first strand of cDNA. Eight target genes (*cat1*, *rhogap*, *tf*, *cyc*, *bro1*, *mic12*, *rpl13*, and *clptm1l*) were selected for expression validation, with the *actin* gene serving as an internal reference for normalization. The primer sequences used for RT-qPCR were listed in **Supplementary Table 1**. RT-qPCR reactions were performed with TB Green^®^ Premix Ex Taq™ II (TaKaRa, Japan) on a QuantStudio™ 6 Flex Real-Time PCR System (Thermo Fisher Scientific, USA). The thermal cycling program consisted of an initial denaturation at 95 °C for 2 min, followed by 40 cycles of 95 °C for 15 s, 55 °C for 20 s, and extension at 72 °C for 60 s. Each treatment group included three independent biological replicates, with three technical replicates run per biological sample to guarantee experimental reproducibility and statistical robustness. The relative expression level of genes was calculated using the 2^−ΔΔCt^ comparative threshold cycle method, and data statistics and graphing were performed.

### 2.10 Data processing and statistical analysis

All experiments were performed with three independent biological replicates All raw numerical data obtained from repeated independent trials were aggregated to calculate the mean values ± standard error for subsequent statistical analysis and visualization. Graph plotting and statistical calculations were conducted using R 4.5.2 and GraphPad Prism 10.1.2; one-way analysis of variance (ANOVA) was first applied to evaluate overall intergroup differences, and Duncan’s new multiple range test was adopted as the *post-hoc* test for pairwise multiple comparisons, with the significance level set at *P* < 0.05 for all analyses.

## 3 Results

### 3.1 Inhibitory effect of limonene on *F. oxysporum*

**Figure 1:**
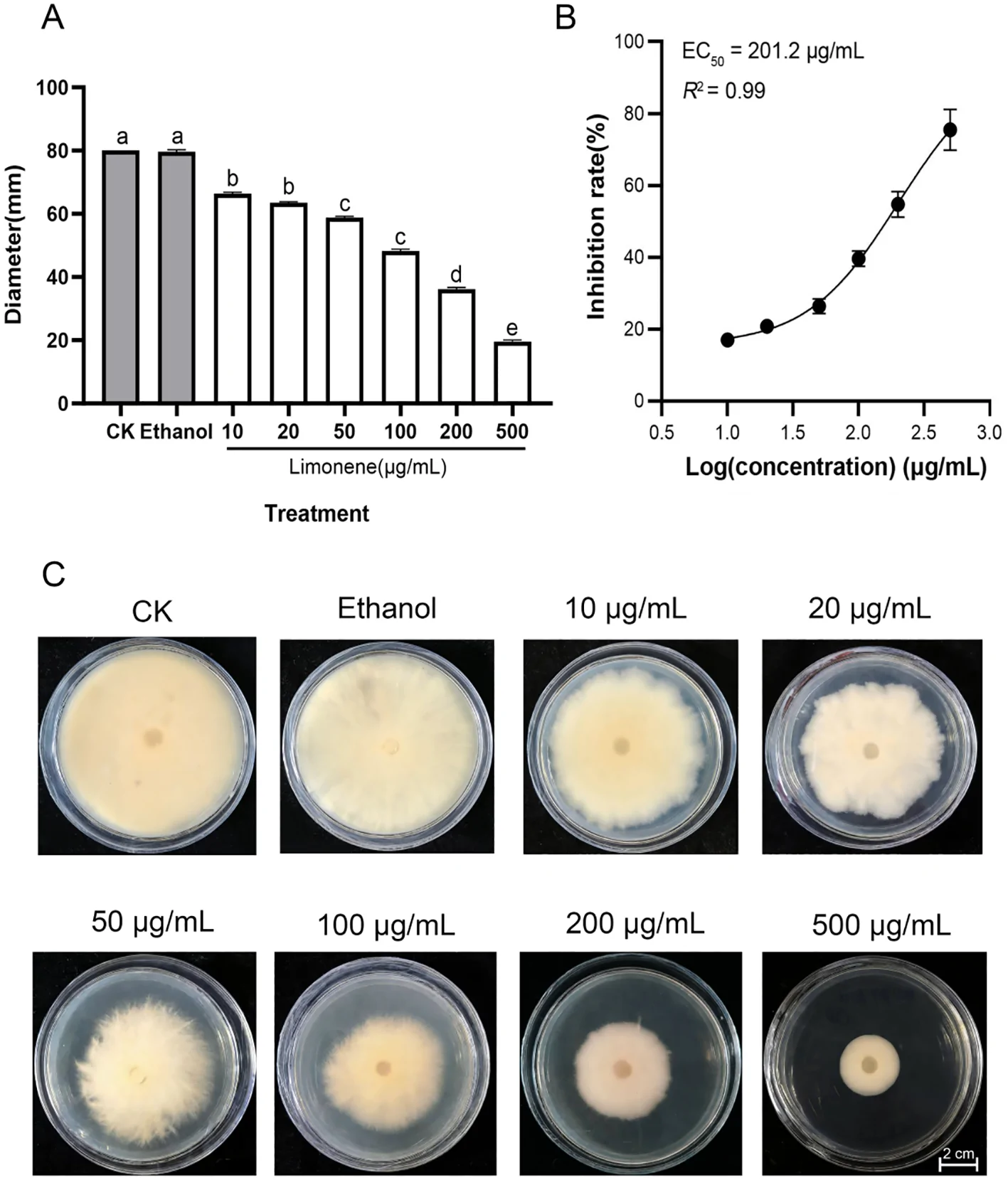
Inhibitory effect of limonene on *F. oxysporum* mycelial growth. **(A)** Colony diameter of *F. oxysporum* treated with limonene. **(B)** Dose-response curve showing inhibition rate, with EC_50_ = 201.2 μg/mL (1.48 mM, *R*^2^ = 0.99). **(C)** Representative colony images. CK: untreated control; Ethanol: 0.5% (v/v) solvent control. Different lowercase letters denote significant differences (*P* < 0.05), n = 3.

The plate antifungal experiment revealed that limonene exerted a significant dose-dependent inhibitory effect on the mycelial growth of *F. oxysporum* (**Figure 1**). No statistical difference in colony diameter was found between blank CK and 0.5% ethanol solvent control (*P* = 0.883). Significant mycelial growth suppression relative to CK was observed starting at 10 μg/mL (0.07 mM) limonene, and this inhibitory effect strengthened sequentially at 20 μg/mL (0.15 mM), 50 μg/mL (0.37 mM), 100 μg/mL (0.73 mM), 200 μg/mL (1.47 mM) and 500 μg/mL (3.67 mM), with all adjusted *P* < 0.001 for every limonene concentration compared to CK. The dose-response analysis yielded an EC_50_ value of 201.2 μg/mL (1.48 mM, *R*^2^ = 0.99), indicating the concentration of limonene required to achieve 50% inhibition of mycelial growth, further confirming its potent antifungal activity against *F. oxysporum*.

### 3.2 Inhibition of *F. oxysporum* mycelial growth by limonene

**Figure 2:**
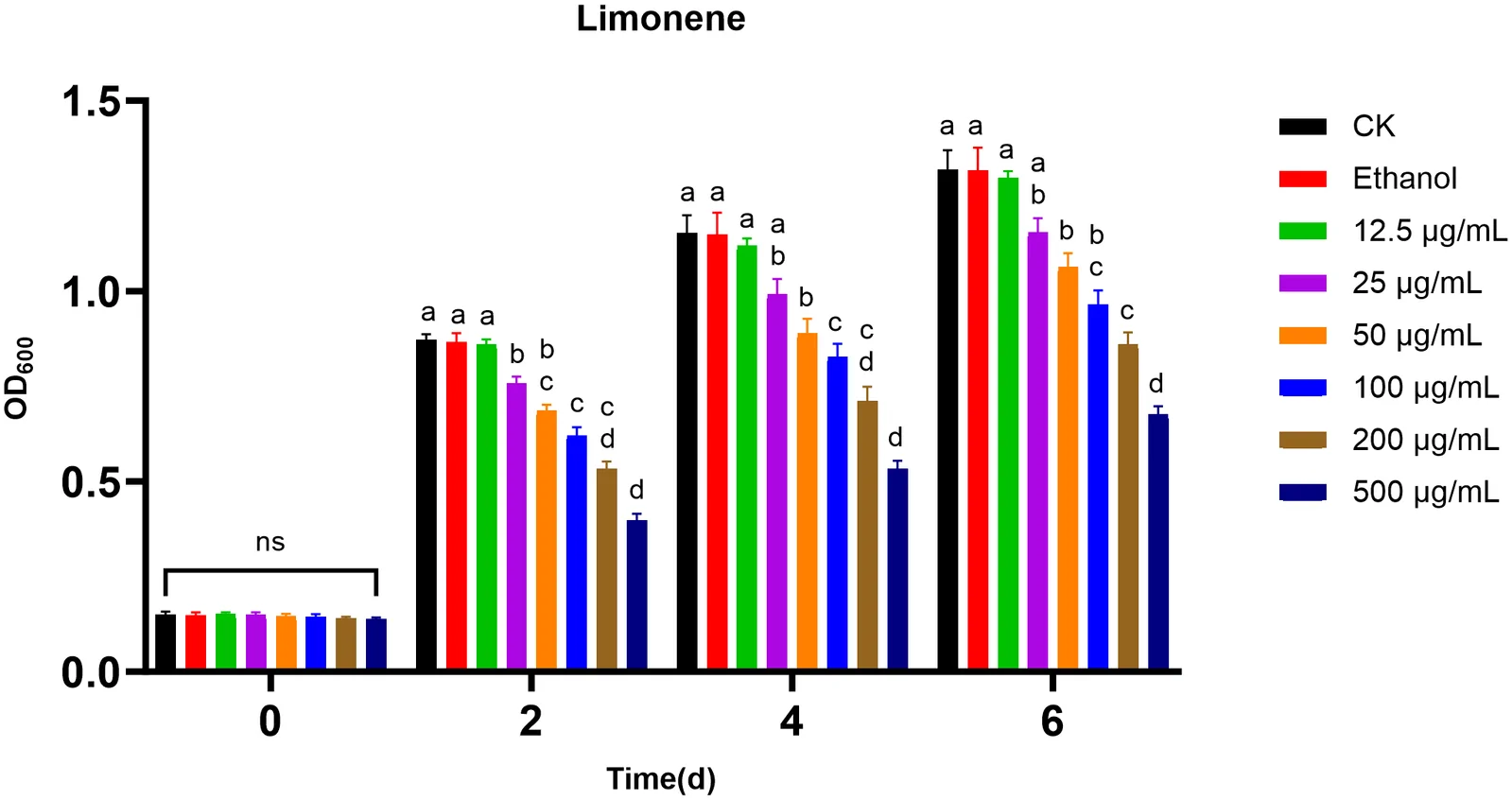
Antifungal activity of limonene against *F. oxysporum*. Tested limonene concentrations: 12.5, 25.0, 50.0, 100.0, 200.0, 500.0 μg/mL (equivalent to 0.09, 0.18, 0.37, 0.73, 1.47, 3.67 mM). CK: untreated control; Ethanol: 0.5% (v/v) solvent control. Different lowercase letters indicate significant differences (*P* < 0.05), n = 3.

The microplate growth assay further verified the dose-dependent antifungal effect of limonene against *F. oxysporum* (**Figure 2**). No significant differences existed between blank CK and ethanol solvent control across all three time points (all *P* > 0.999). At 2 d, 4 d and 6 d post-incubation, the 12.5 μg/mL (0.09 mM) group exhibited no statistical difference relative to CK (2 d: *P* = 0.859; 4 d: *P* = 0.785; 6 d: *P* = 0.938), indicating low-dose limonene exerted negligible inhibitory influence on fungal proliferation. In contrast, limonene significantly suppressed pathogen growth and reproduction at all time points starting from 50 μg/mL (2 d: *P* < 0.001; 4 d: *P* = 0.007; 6 d: *P* = 0.011), with inhibitory efficacy strengthening in a concentration-dependent manner and peaking at the maximum tested concentration of 500 μg/mL (3.67 mM).

### 3.3 Effect of limonene on the growth chemotaxis of *F. oxysporum*

**Figure 3:**
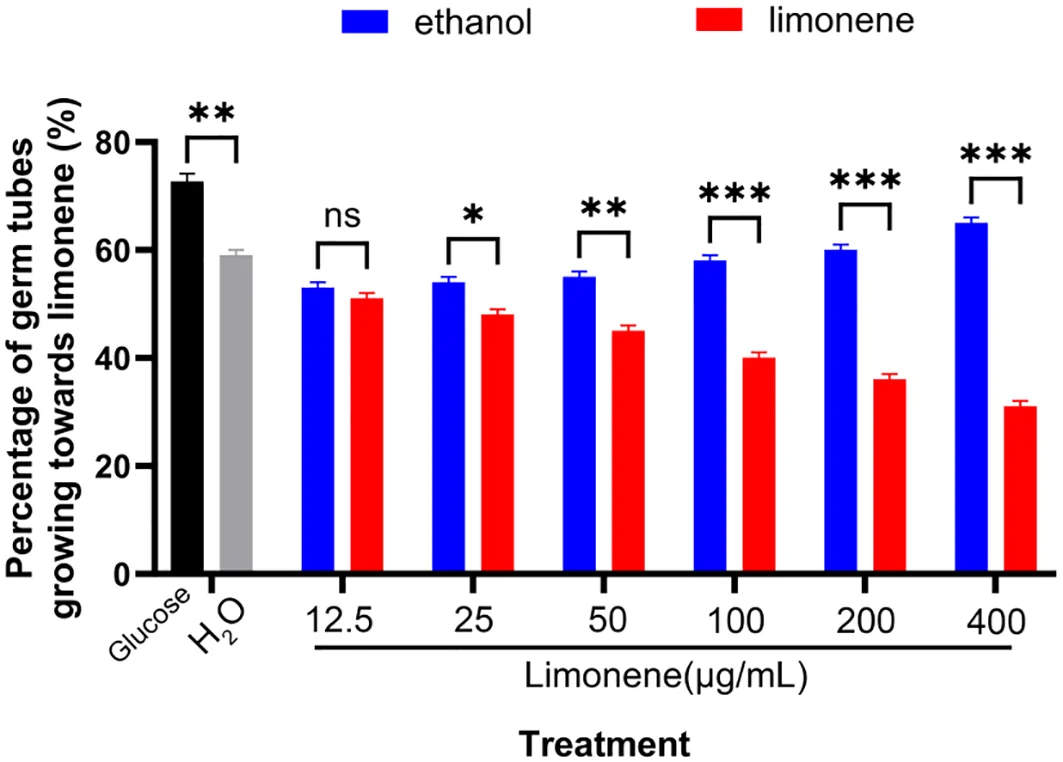
Chemotactic effect of limonene on *F. oxysporum* germ tubes. Glucose was used as the positive control. Asterisks indicate significant differences compared with the ethanol group (*P < 0.05, **P < 0.005, ***P < 0.001; ns, not significant).

The results of the chemotaxis experiment indicated that treatment with 25 μg/mL (0.18 mM, *P* = 0.01) limonene resulted in a greater number of *F. oxysporum* mycelia growing towards ethanol compared to limonene (**Figure 3**). As the concentration of limonene increased, the number of mycelia growing towards it consistently decreased, with a significant inhibitory effect observed at higher concentrations. The mycelia count reached its minimum at concentrations of 200 and 400 μg/mL (1.47 and 2.94 mM), suggesting that high concentrations of limonene exert a strong inhibitory or repellent effect on the chemotactic behavior of *F. oxysporum*, with the inhibitory effect intensifying as the concentration increases.

### 3.4 Effect of limonene on the integrity and permeability of the mycelial cell membrane of *F. oxysporum*

**Figure 4:**
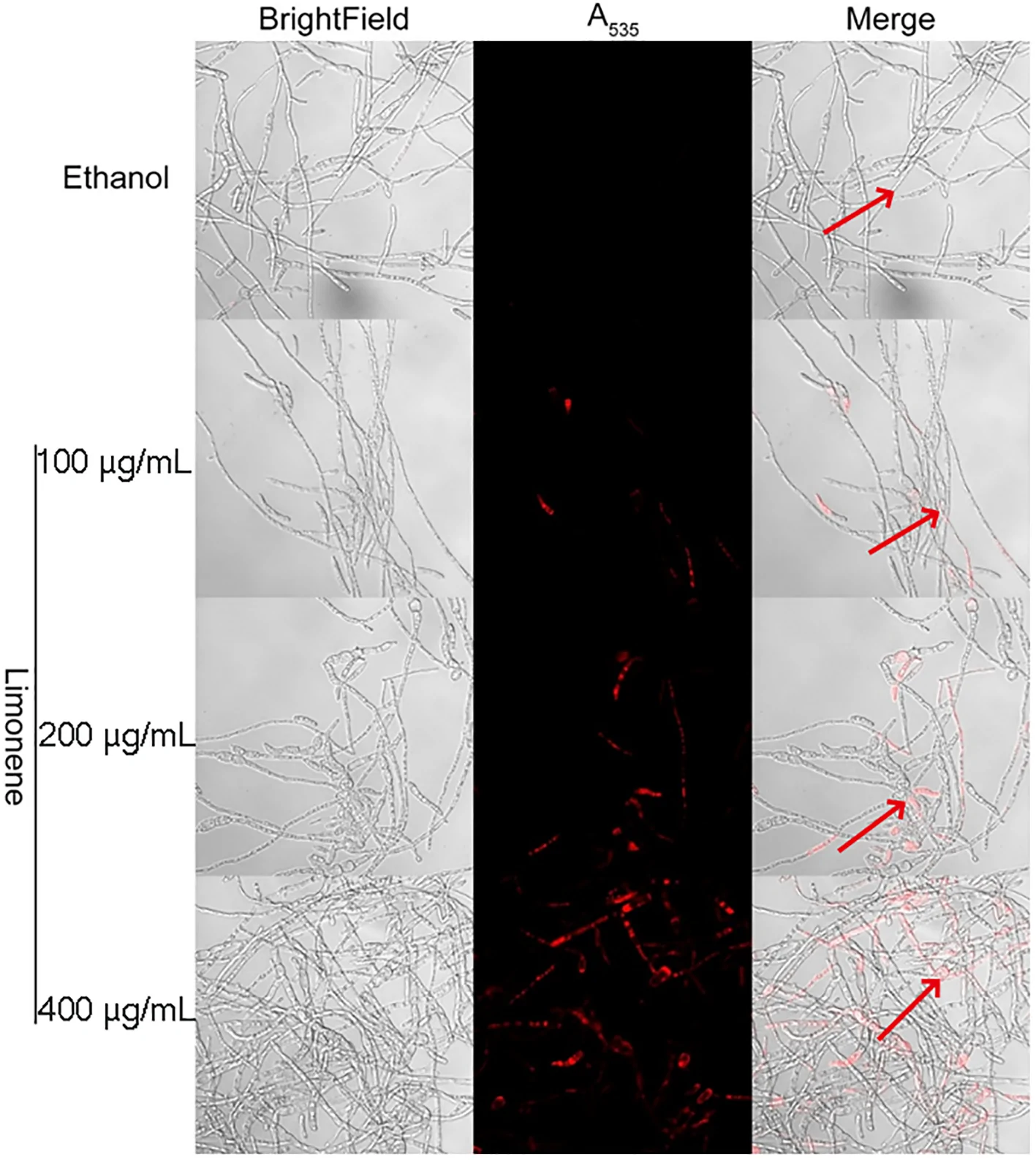
PI staining for *F. oxysporum* hyphal membrane integrity. Bright field, PI fluorescence (A_535_) and merged channels are shown. Treatments: 0.5% (v/v) ethanol control, limonene at 100.0, 200.0, 400.0 μg/mL (0.73, 1.47, 2.94 mM). Red solid arrows in merged panels enclose typical hyphal areas to display concentration-dependent membrane damage.

PI staining observations indicated that hyphae in the ethanol control group remained intact, exhibiting no discernible red fluorescence, which suggests that the cell membranes were preserved (**Figure 4**). In contrast, treatment with 100 μg/mL (0.73 mM) limonene resulted in weak fluorescence, while the groups treated with 200 μg/mL (1.47 mM) and 400 μg/mL (2.94 mM) exhibited strong and intense red fluorescence. These findings suggested that limonene disrupted the integrity of the cell membranes in *F. oxysporum* in a concentration-dependent manner.

**Figure 5:**
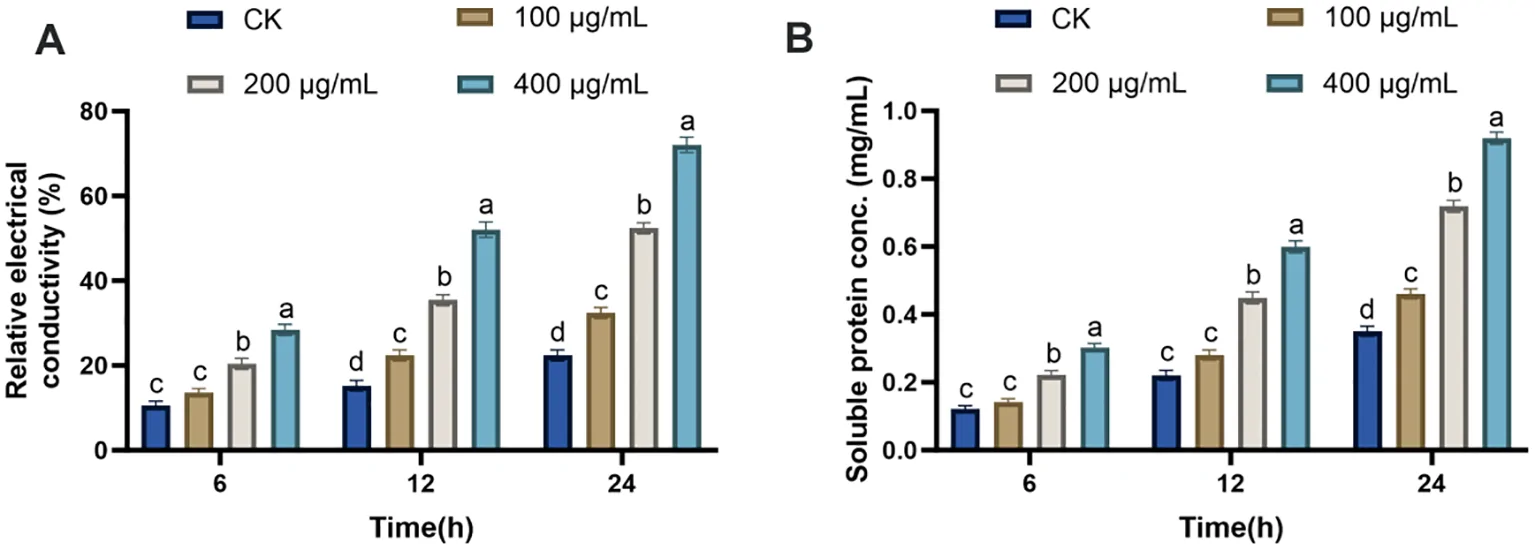
Changes in extracellular relative conductivity **(A)** and soluble protein content **(B)** of *F. oxysporum* mycelia after 6, 12 and 24 h exposure to limonene at 100.0 μg/mL (0.73 mM), 200.0 μg/mL (1.47 mM) and 400.0 μg/mL (2.94 mM). CK: untreated control group. Different lowercase letters a–d indicate significant differences among treatments at the same time point (*P* < 0.05).

To further confirm the detrimental effect of limonene on the cell membrane, we measured the changes in relative electrical conductivity and protein content in the supernatant after treatment with limonene. At 6, 12, and 24 h, the group treated with 400 μg/mL (2.94 mM) limonene exhibited the highest relative electrical conductivity, followed by the 200 μg/mL (1.47 mM) and 100 μg/mL (0.73 mM) groups, as well as the control (CK) group (**Figure 5A**). A similar trend was observed in the soluble protein concentration (**Figure 5B**). At 24 h, the 400 μg/mL (2.94 mM) limonene treatment group reached the highest level of soluble protein concentration, followed by the 200 μg/mL (1.47 mM), 100 μg/mL (0.73 mM), and CK groups, indicating that higher concentrations of limonene and longer treatment durations promoted the leakage of intracellular components in *F. oxysporum*.

### 3.5 Transcriptomic analysis of *F. oxysporum* after limonene treatment

**Figure 6:**
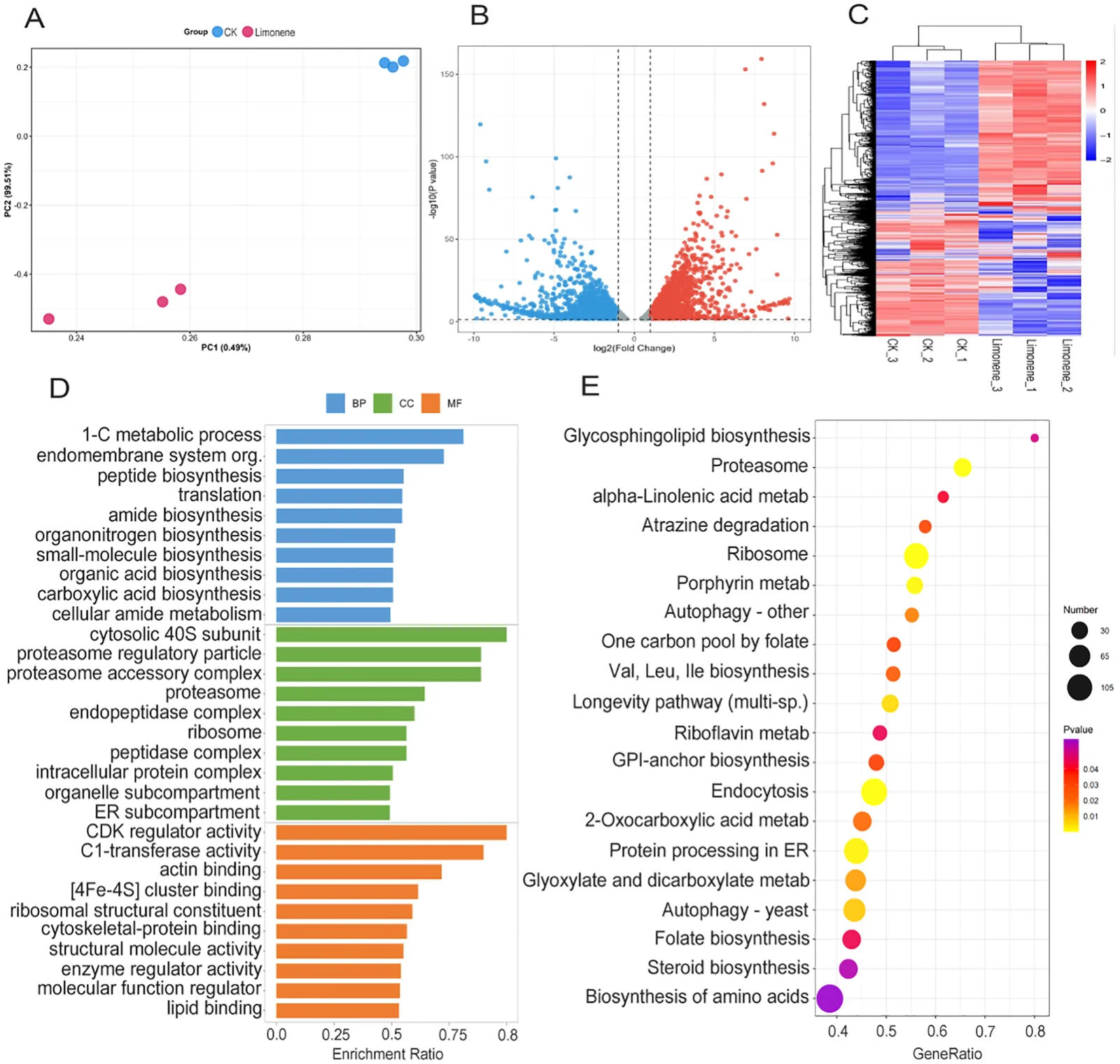
Transcriptome analysis of *F. oxysporum*. **(A)** PCA; **(B)** Volcano plot of DEGs; **(C)** DEG heatmap; **(D)** GO enrichment analysis; **(E)** KEGG pathway enrichment analysis.

To further explore the antifungal mechanism of limonene on *F. oxysporum*, we employed transcriptomics to identify the core functional genes of *F. oxysporum*. The transcriptomic sequencing generated 38.06 Gbp of clean data following quality control. The corresponding raw data have been deposited in the NCBI SRA database under bioproject accession number PRJNA1438381. The PCA results showed that the samples of the control group and the limonene treatment group were completely separated in the dimensions of PC1 (0.49%) and PC2 (99.51%), with the intra-group replicates closely clustered, thereby verifying the experimental reproducibility and confirming that limonene significantly reshaped the transcriptomic characteristics of the pathogen (**Figure 6A**). A total of 3871 DEGs were identified with |log_2_FC| ≥ 1 and *P* < 0.05, including 1973 upregulated and 1898 downregulated genes (**Figure 6B**, **Supplementary Table 2**). Significantly upregulated DEGs included key functional genes such as catalase P1, major facilitator superfamily domain-containing protein, and RmlC-like cupin domain-containing protein, which were potentially involved in stress response, substrate transport, and secondary metabolism of *F. oxysporum* under limonene treatment. In contrast, significantly downregulated DEGs mainly comprised uncharacterized protein, glycoside hydrolase superfamily, and general substrate transporter, indicating that limonene may inhibit fungal growth and metabolism by suppressing carbohydrate hydrolysis and nutrient transport-related genes. The unsupervised hierarchical clustering heatmap of DEGs demonstrated that the two groups of samples formed distinct clusters based on the expression profiles, exhibiting high intra-group consistency and significant inter-group differences (**Figure 6C**).

The GO functional enrichment analysis examined the functional distribution of DEGs from three perspectives: biological process, cellular component, and molecular function (**Figure 6D**, **Supplementary Table 3**). At the cellular component level, cytosolic small ribosomal subunit and proteasome-related complexes accounted for the highest enrichment ratio; at the molecular function level, functions such as cyclin-dependent protein serine/threonine kinase regulator activity, hydroxymethyl, formyl and related transferase activity, actin binding, 4 iron, 4 sulfur cluster binding, and structural constituent of ribosome were highly enriched; at the biological process level, pathways such as one-carbon metabolic process, peptide biosynthetic process, translation, and organonitrogen compound biosynthetic process were significantly enriched. This indicates that limonene mainly regulates the growth and stress response of pathogens by interfering with protein synthesis, one-carbon metabolism, organonitrogen biosynthesis, and basic catalytic metabolic processes.

KEGG pathway enrichment analysis was conducted to elucidate the core metabolic and signaling pathways associated with DEGs. The results indicated that the proteasome, ribosome, and alpha-Linolenic acid metabolism pathways demonstrated a high rich factor, gene count, and significant enrichment (**Figure 6E**, **Supplementary Table 4**). Furthermore, pathways such as protein processing in endoplasmic reticulum, autophagy-yeast, autophagy-other, endocytosis, glyoxylate and dicarboxylate metabolism, and glycosphingolipid biosynthesis-ganglio series were also significantly enriched. These findings suggested that limonene might coordinately disrupt protein synthesis, translation, degradation and autophagy systems, as well as fatty acid metabolism, sphingolipid biosynthesis, one-carbon metabolism and stress responses in the pathogen, ultimately inhibiting its physiological functions and growth.

### 3.6 RT-qPCR validation analysis

**Figure 7:**
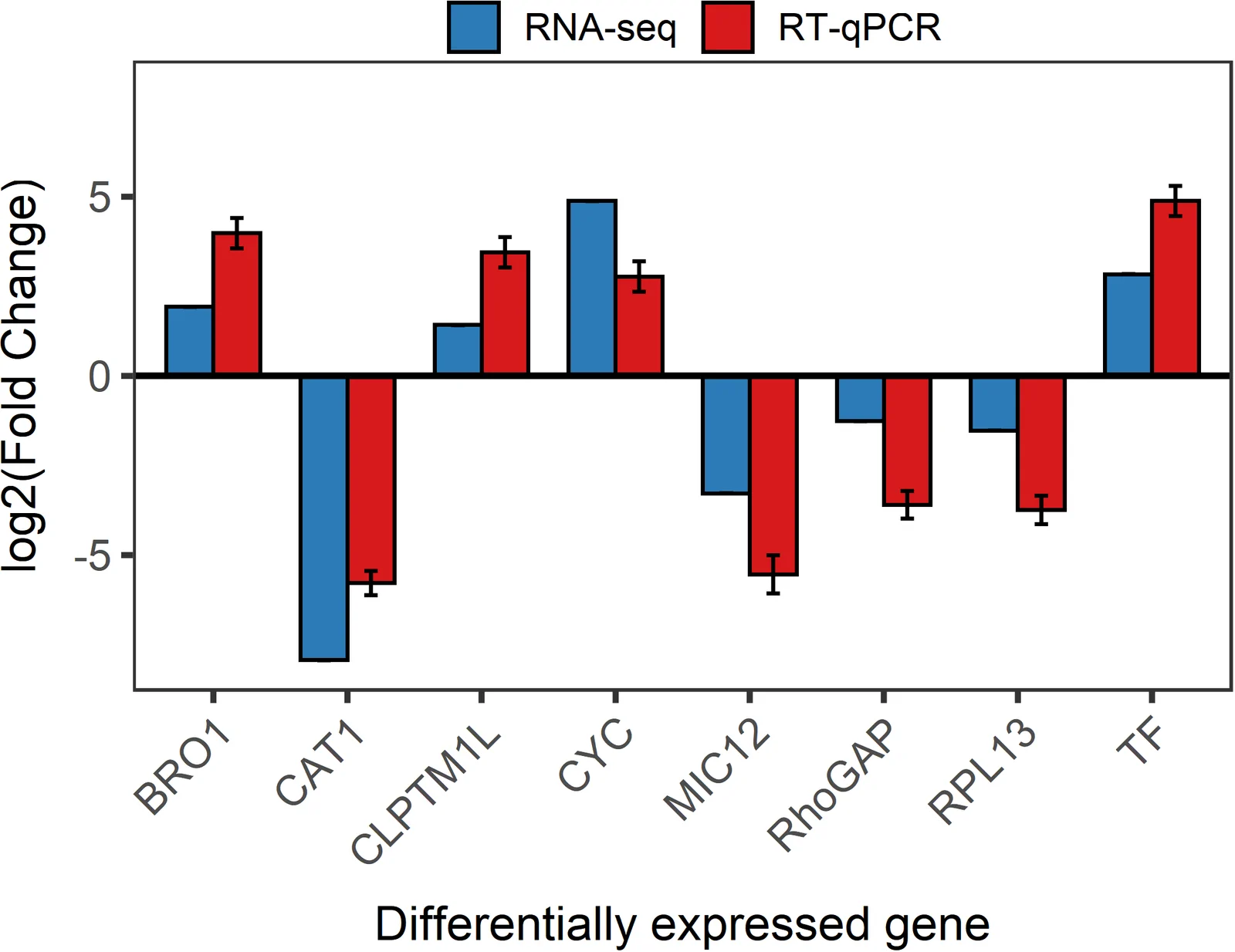
RT-qPCR validation of eight key DEGs in *F. oxysporum* under limonene treatment. BRO1 (*bro1*), BRO1-like domain-containing protein; CAT1 (*cat1*), catalase P1; CLPTM1L (*clptm1l*), CLPTM1L-like protein; CYC (*cyc*), cyclin family protein; MIC12 (*mic12*), MICOS complex subunit Mic12 family protein; RhoGAP (*rhogap*), Rho GTPase-activating protein; RPL13 (*rpl13*), 60S ribosomal protein eL13; TF (*tf*), transcription factor domain-containing protein.

We also noted that limonene treatment significantly regulated a set of core metabolic and signaling pathways, including glyoxylate and dicarboxylate metabolism, longevity regulating pathway-multiple species, protein processing in endoplasmic reticulum, autophagy-yeast, endocytosis, porphyrin metabolism, ribosome, and proteasome. A total of eight pivotal differentially expressed genes (DEGs) involved in these pathways exhibited pronounced expression alterations (**Figure 7**).

In the glyoxylate and dicarboxylate metabolism pathway (ko00630), the gene encoding catalase P1 (*cat1*) was significantly downregulated with an expression fold change of 0.004. The gene *rhogap*, which encodes the Rho GTPase-activating protein (RhoGAP) assigned to the longevity regulating pathway-multiple species (ko04213), was downregulated to 0.42-fold. The transcription factor-encoding gene *tf*, whose product (TF) participates in protein processing in endoplasmic reticulum (ko04141), was significantly upregulated by 7.14-fold. Within the autophagy-yeast pathway (ko04138), the cyclin gene *cyc* (protein product CYC) was remarkably upregulated by 29.51-fold. The gene *bro1* encoding BRO1-like domain-containing protein (BRO1) in the endocytosis pathway (ko04144) displayed a significant upregulation of 3.81-fold. In the porphyrin metabolism pathway (ko00860), *mic12*, coding for the MICOS complex subunit Mic12 family protein (MIC12), was markedly downregulated to 0.10-fold. The ribosomal gene *rpl13* encoding 60S ribosomal protein eL13 (RPL13), belonging to the ribosome pathway (ko03010), showed a decreased expression level of 0.35-fold. Moreover, *clptm1l*, the gene for CLPTM1L-like protein (CLPTM1L) in the proteasome pathway (ko03050), was significantly upregulated by 2.68-fold.

Furthermore, the expression patterns of these eight DEGs determined by RT-qPCR were highly consistent with the RNA-seq results. Collectively, these findings demonstrated that limonene treatment orchestrates multiple metabolic and signaling pathways to disrupt key physiological processes in *Fusarium oxysporum*.

### 3.7 Metabolomic analysis of *F. oxysporum* after limonene treatment

**Figure 8:**
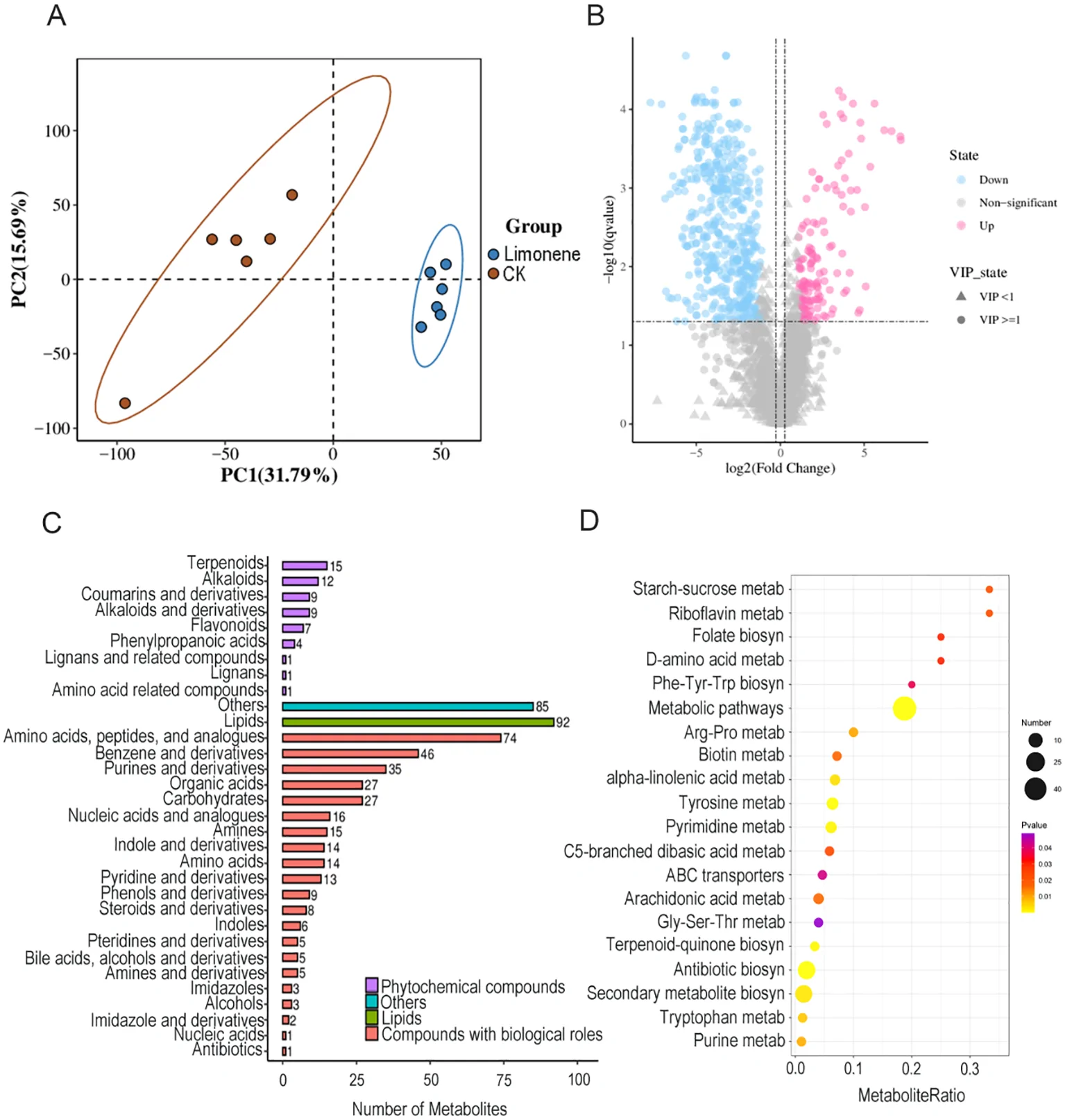
Metabolome analysis of *F. oxysporum* under limonene treatment. **(A)** PLS-DA; **(B)** Differential metabolite volcano plot; **(C)** Classification of identified metabolites; **(D)** KEGG enrichment.

To explore the regulatory effect of limonene on the metabolome of *F. oxysporum*, we first evaluated the global metabolomic differences using partial least squares discriminant analysis (PLS-DA). The PLS-DA results showed that the samples of the blank control group and the limonene treatment group were clearly separated in the dimensions of PC1 (31.79%) and PC2 (15.69%), with good intra-group replicate clustering. This confirmed that limonene significantly changed the metabolomic characteristics of the pathogen (**Figure 8A**). A total of 9317 metabolites were identified in the positive and negative ion modes, among which 626 differential accumulated metabolites (DAMs) were identified with |log_2_FC| ≥ 1 and q-value < 0.05. This included 133 upregulated and 493 downregulated metabolites, with the number of downregulated metabolites significantly exceeding that of upregulated ones (**Figure 8B**; **Supplementary Tables 5**, **S6**). The classification of DAMs illustrated the chemical class distribution of metabolites in limonene-treated *F. oxysporum*, with lipids being the most abundant category, followed by others. In the category of phytochemical compounds, terpenoids and alkaloids were the most enriched subclasses. Particularly, three core differential metabolites, ortho-Hydroxyphenylacetic acid, 4-Methylphenol, and Lenticin, exhibited significant abundance changes after limonene treatment. Among the compounds with biological roles, amino acids, peptides, and analogues, benzene and derivatives, as well as purines and derivatives represented the most numerous subclasses (**Figure 8C**).

KEGG pathway enrichment analysis revealed that DAMs were significantly enriched in pathways such as starch and sucrose metabolism, riboflavin metabolism, folate biosynthesis (**Figure 8D**, **Supplementary Table 7**). Remarkably, starch and sucrose metabolism, riboflavin metabolism exhibited high enrichment factors and low P-values, reflecting robust enrichment significance. Collectively, these results indicated that limonene primarily disturbed carbohydrate metabolism and cofactor biosynthesis in *F. oxysporum* by modulating these core metabolic pathways.

### 3.8 Integrated omics analysis of *F. oxysporum* after limonene treatment

**Figure 9:**
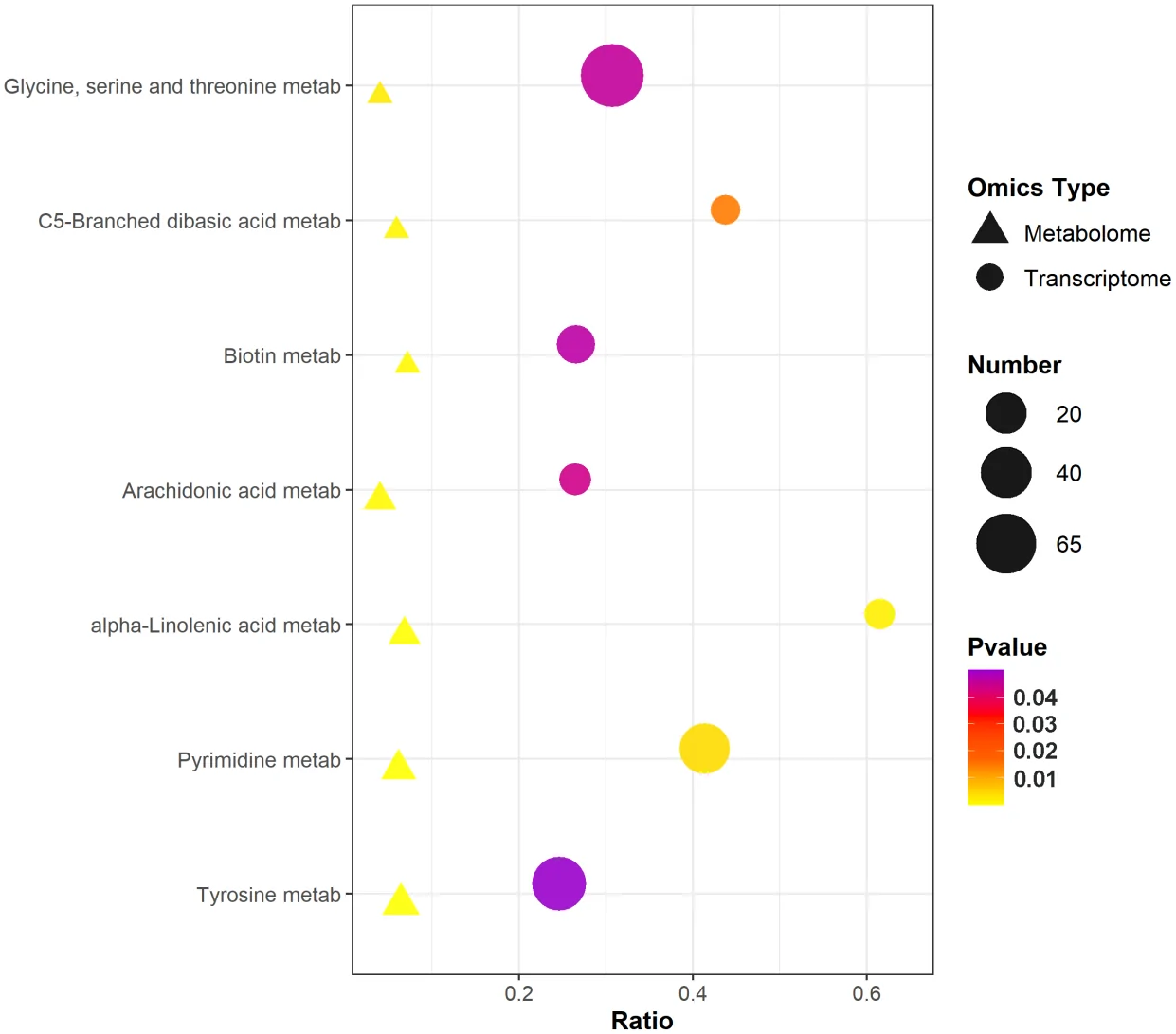
Integrated transcriptomic and metabolomic KEGG enrichment analysis in response to limonene treatment.

Finally, we integrated the transcriptomic and metabolomic results with metabolic pathways as the framework and conducted joint mapping analysis relying on differentially expressed genes (DEGs) and differentially accumulated metabolites (DAMs). KEGG enrichment identified seven significantly perturbed pathways modulated by both DEGs and DAMs in limonene-treated *F. oxysporum*, including arachidonic acid metabolism, tyrosine metabolism, biotin metabolism, glycine, serine and threonine metabolism, pyrimidine metabolism, C5-branched dibasic acid metabolism, and alpha-linolenic acid metabolism (**Figure 9**).

Among these seven pathways, alpha-linolenic acid metabolism was the only pathway that exhibited the most significant changes. In this pathway, eight DEGs were identified, comprising five that were upregulated and three that were downregulated. Additionally, five related lipid intermediates were also altered, all of which were downregulated. These changes directly impacted membrane lipid metabolism and oxidation processes in *F. oxysporum*.

In the tyrosine metabolism pathway, key catalytic enzymes and aromatic amino acid intermediates were significantly varied in response to limonene treatment. Related synthetases and dehydrogenases were notably downregulated, whereas several catabolic genes were observably upregulated, leading to unbalanced accumulation of downstream metabolites. In the glycine, serine and threonine metabolism pathway, genes encoding amino acid-related synthases and transferases were coordinately expressed, leading to corresponding fluctuations in amino acid metabolites. Pathways including arachidonic acid metabolism and biotin metabolism also exhibited coordinated changes in genes and metabolites, which jointly participated in the stress response of *F. oxysporum* under limonene exposure.

## 4 Discussion

This study systematically investigated the inhibitory effect and molecular mechanism of limonene on the tobacco root rot pathogen *F. oxysporum* through antifungal experiments, physiological detection combined with transcriptomic and metabolomic multi-omics technologies. It was confirmed that limonene had a significant dose-dependent inhibitory effect on this pathogen, with its antifungal action attributed to the destruction of the pathogen’s cell membrane structure, the remodeling of the transcriptional regulatory network, and multiple interference with metabolic pathways.

Many studies have documented the inhibitory potential of limonene against *F. oxysporum*. For instance, Cruz et al. (2024) revealed that limonene-rich orange peel wax extract exhibited prominent antifungal efficacy against *F. oxysporum* at a concentration of 50 mg/mL (367.03 mM). Similarly, Xia and Dong (2025) demonstrated that limonene effectively inhibited the colony expansion of the potato dry rot pathogen *F. oxysporum*, with an IC_50_ of 8.32 μL/mL (51.36 mM), significantly higher than the EC_50_ of 201.2 μg/mL (1.48 mM) determined in the present study. However, Marei et al. (2012) reported an EC_50_ of 153.2 mg/L (1.13 mM) for (S)-limonene against *F. oxysporum*, which was lower than our measured value. Such discrepancies in reported efficacy values may be attributed to the distinct pathogenic specializations of fungal strains and the different optical isomers of limonene.

To comprehensively evaluate the application potential of limonene, we further compared its antifungal efficacy with that of commercial synthetic fungicides against *Fusarium* spp. Prochloraz, an imidazole antifungal agent, exhibited the highest antifungal efficiency with an EC_50_ of 0.07 mg/L (0.19 mM) against the pepper pathogen *F. oxysporum*. In contrast fluopyram, a broad-spectrum fungicide, displayed relatively weak inhibitory activity, with an EC of 1075.01 mg/L (3.71 mM) against the same pathogen (Mihajlovic et al., 2021). Prothioconazole, a triazinone fungicides, showed potent activity against *F. pseudograminearum* with an average EC_50_ of 0.61 μg/mL (1.77 mM) (He et al., 2025). Additionally, triazole fungicides (metconazole, prothioconazole, and tebuconazole) exhibited inhibitory effects on the *F. graminearum* species complex, with EC values ranging from 0.09 to 23.6 mg/L (Matelionienė et al., 2024). Comparative analysis revealed that the limonene EC_50_ determined in this study (201.2 μg/mL, 1.48 mM) falls within the effective concentration range of commercial fungicides against *Fusarium* spp., showing considerably stronger inhibitory activity against *F. oxysporum* than fluopyram but weaker efficacy than high-efficiency triazole fungicides including prochloraz and prothioconazole. Notably, synthetic fungicides often exhibit inconsistent *in vitro* and *in vivo* performance: azoxystrobin exerts satisfactory *in vitro* mycelial inhibition but poor greenhouse disease control efficacy, whereas captan achieves an *in vivo* disease control rate as high as 95.60% (Mihajlovic et al., 2021). Considering these significant discrepancies between laboratory and field efficacy, further greenhouse verification and field trials are indispensable to validate the practical biocontrol potential and field application value of limonene for the management of *F. oxysporum*-caused TRR.

Chemotaxis refers to the directional movement of motile microorganisms along a chemical concentration gradient in response to attractants or repellents (Adler, 1966). In the context of pathogen infection, chemotaxis plays a pivotal role in the entire pathogenic process: pathogens rely on chemotactic signal transduction systems to sense chemical cues, thereby efficiently locating, adhering to, and colonizing host mucosal surfaces and target tissues, which is a prerequisite for successful invasion and subsequent proliferation and toxin secretion (Matilla and Krell, 2017). Consequently, interfering with pathogen tropism or inducing negative chemotaxis can prevent pathogens from colonizing the host plant, thereby achieving the goal of disease resistance. In this study, limonene exhibited high-concentration chemotactic inhibition against *F. oxysporum*, with no low-concentration attractive effect observed. This finding was distinct from previous findings reported by Yang et al. (2022), who demonstrated a concentration-dependent dual regulatory effect of D-limonene on the chemotaxis of *Phytophthora capsici* zoospores: attraction at 10 μg/mL (0.07 mM) and inhibition at 100 μg/mL (0.73 mM). The divergent result was speculated to stem from two factors: first, differences in pathogen classification—*Phytophthora* (Oomycota) could recognize low-concentration limonene as a chemotactic signal, whereas the signal recognition system of *F. oxysporum* (Ascomycota) showed no such response; second, variations in experimental systems—this study involved *in vitro* treatment with pure limonene alone, lacking the synergistic effects of other plant terpenoids.

The disruption of cell membrane integrity is a key mechanism by which natural products inhibit *F. oxysporum*. Plant-derived terpenoid compounds exert prominent antifungal activity against *F. oxysporum* through damaging membrane structure and increasing membrane permeability, resulting in the leakage of intracellular substances (Zhou et al., 2024). In this study, we observed an increase in extracellular electrical conductivity and soluble protein content over time and with increasing concentration following limonene treatment, which contributed to the disruption of cellular metabolic homeostasis. Burt (2004) noted that the hydrophobicity of limonene enabled it to integrate into the lipid bilayer of the cell membrane, resulting in membrane permeabilization and content leakage. Furthermore, Han et al. (2019) confirmed its destructive effects on fungal cell walls and overall cell integrity through scanning electron microscopy, which aligns with the findings of this study.

Multi-omics integration has become a core strategy to decipher the antifungal mechanisms of natural products. Essentially, it performs coupling analysis of gene expression changes (transcriptomics) and phenotypic metabolite abundance (metabolomics), thereby breaking through the “black box” limitation of single omics and revealing the complete regulatory network from molecular targets to physiological phenotypes (Karalija et al., 2025; Kimotho and Maina, 2024). The results of the transcriptomic GO functional enrichment analysis indicated that the GO terms associated with ribosome structural composition, protein translation processes, and substance synthesis were significantly regulated. Concurrently, ATP binding activity and transferase activity were observed to decrease. Similar molecular regulatory characteristics were reported by Wang et al. (2025), who found that walnut green husk extract disrupted microbial translation processes and intracellular energy metabolism, resulting in reduced cellular ATP levels and ATPase activity. KEGG pathway analysis further revealed that the pathways related to protein synthesis and degradation exhibited the most significant regulation following limonene treatment. For example, most DEGs involved in the ribosome pathway were downregulated (10 upregulated DEGs and 87 downregulated DEGs), while the proteasome pathway was upregulated (30 upregulated DEGs and 6 downregulated DEGs). Our qRT-PCR results corroborated these findings. These results aligned with a previous study by Zhou et al. (2025), which indicated that D-limonene inhibited the expression of ribosomal genes by reducing histone acetylation (H3K9ac/H3K27ac), thereby disrupting cytoplasmic translation in *F. proliferatum*. Moreover, Zhang et al. (2024) also found that the ribosome was the most severely affected pathway in fungi exposed to another antifungal essential oil, eugenol, leading to the disruption of the fungal protein synthesis machinery. Collectively, these findings indicated that essential oils exert antifungal effects mainly by disrupting fungal protein synthesis and degradation. At the metabolic level, limonene also triggered drastic shifts in three endogenous fungal metabolites: ortho-Hydroxyphenylacetic acid, 4-Methylphenol, and Lenticin. Endogenous phenolic and defensive secondary metabolites exert dose-dependent autotoxicity on *Fusarium* when intracellular accumulation exceeds physiological tolerance (Viñas and Karlovsky, 2025). This metabolic perturbation well explained the disrupted membrane function and impaired protein synthesis observed in this study. Taken together, transcriptional repression and metabolic autotoxicity jointly mediate the multi-target antifungal effect of limonene on *F. oxysporum*.

In addition, both transcriptomics and metabolomics analyses demonstrated that limonene treatment regulated key energy and amino acid metabolic pathways, including glycolysis/gluconeogenesis, the tricarboxylic acid cycle, and tyrosine metabolism, as well as glycine, serine, and threonine metabolism. The blockade of energy metabolism pathways directly resulted in insufficient ATP synthesis in the pathogen, which hindered energy support for physiological processes such as cell division, substance synthesis, and stress responses (Ene et al., 2014). Disruption of amino acid metabolism further obstructed protein synthesis, adversely affecting the production of enzymes and structural proteins and aggravating metabolic pathway abnormalities (Boyce et al., 2015). Jian et al. (2023) reported similar findings, noting that limonene inhibited the growth of *Fusarium graminearum* and the production of DON by obstructing metabolic pathways such as galactose metabolism, glycolysis/glyconeogenesis, glyoxylate dicarboxylate metabolism, and amino acid metabolism. Notably, Jian’s research focused on grain-sourced *F. graminearum*, whereas our target pathogen was isolated from diseased tobacco roots, representing a distinct host-adapted *Fusarium* pathotype for testing.

Concurrently, the significant perturbation of alpha-linolenic acid metabolism introduces a devastating dimension of membrane disintegration and lipid signaling failure. Alpha-linolenic acid, a vital polyunsaturated fatty acid (PUFA), is not only a fundamental component of fungal phospholipid bilayers that maintain membrane fluidity and structural integrity, but also serves as the precursor for synthesizing oxylipins—critical lipid-derived signaling molecules that govern fungal development and pathogenicity (Beccaccioli et al., 2022; Veloso and Díaz, 2021; Zhu et al., 2024). The disruption of this pathway indicated profound damage to membrane architecture and a breakdown in oxylipin-mediated communication. Collectively, limonene executed a lethal “multiple-punch” strategy: it collapsed the central energy supply and dismantles the physical and chemical signaling networks via alpha-linolenic acid pathway disruption. This multi-pronged attack culminates in irreversible physiological disintegration and the complete loss of pathogenicity.

While previous multi-omics analyses of limonene mainly centered on disturbances within protein and energy metabolic cascades across different *Fusarium* isolates, our integrated transcriptomic and metabolomic profiling captured synchronous shifts in alpha-linolenic acid metabolism at both transcriptional and metabolite levels. The core biological function of this lipid pathway in sustaining fungal membrane integrity and mediating oxylipin-dependent pathogenic signals has been fully validated (Beccaccioli et al., 2022; Veloso and Díaz, 2021; Zhu et al., 2024). While all the above molecular regulatory patterns were characterized under *in vitro* pure culture, further validation under complex soil and cultivation conditions is required to confirm whether such inhibitory effects persist in realistic agricultural scenarios.

This study still has certain limitations. All experiments were only conducted under *in vitro* culture. As summarized by Ibáñez et al. (2020), limonene is highly volatile and easily degraded under light and temperature, restricting its field efficacy; we did not carry out encapsulation and formulation optimization to improve its environmental stability. Besides, we only tested single limonene without exploring synergistic effects with other terpenoids. In addition, this work lacks evaluation of limonene’s phytotoxicity to tobacco seedlings, which varies with plant species at high concentrations (Ibáñez et al., 2020), as well as its influences on rhizosphere beneficial microbes, failing to meet the complete ecological safety assessment standards for botanical pesticides proposed by Han et al. (2019). Based on this, future research should focus on three aspects: first, develop stabilized limonene formulations, optimize formulation design and controlled-release technology to improve its field stability; second, explore the synergistic antifungal mechanism of limonene with other natural active ingredients to improve the overall prevention and control effect; third, comprehensively evaluate the impact of limonene on soil microbial communities, non-target organisms, and the ecological environment to clarify its environmental safety; at the same time, the limonene synthesis ability of tobacco itself can be improved through genetic engineering methods to provide new strategies for the green and sustainable prevention and control of tobacco root rot.

## 5 Conclusions

This study confirmed that limonene exhibits a significant inhibitory effect on tobacco-derived *F. oxysporum*, making it a viable candidate for the prevention and control of tobacco root rot. A potential mechanism of action is speculated to primarily involve the disruption of the integrity of the fungal cell membrane, which interferes with various processes, including energy metabolism, amino acid metabolism, lipid metabolism, and protein synthesis and degradation. Consequently, this disruption may contribute to inhibiting the growth, proliferation, and chemotactic behavior of the pathogen. The findings of this study provide a crucial theoretical foundation for the environmentally friendly prevention and control of tobacco root rot, and suggest the promising application potential of limonene as a botanical fungicide, with further mechanistic verification required.

## Data Availability

The data presented in the study are deposited in the NCBI Sequence Read Archive (SRA) repository, accession number PRJNA1438381. The other data presented in the study are deposited in the article and Supplementary Material, with relevant supporting details available in the article/**Supplementary Material**.

## References

[B1] Abdel-Kawy M. A. Michel C. G. Kirollos F. N. Hussien R. A. A. Al-Mahallawi A. M. Sedeek M. S. (2021). Chemical composition and potentiation of insecticidal and fungicidal activities of Citrus trifoliata L. fruits essential oil against Spodoptera littoralis, Fusarium oxysporum and Fusarium solani via nano-cubosomes. Nat. Prod. Res. 35, 2438–2443. doi: 10.1080/14786419.2019.1675063 31596140

[B2] Adler J. (1966). Chemotaxis in bacteria. Science 153, 708–716. doi: 10.1126/science.153.3737.708 4957395

[B3] Almjalawi B. Chechan R. Al-hadedee L. (2023). Using food residues (potato peels) as an alternative to potato dextrose agar in the growth of edible food fungi. J. Appl. Natural Sci. 15, 211–219. doi: 10.31018/jans.v15i1.3989

[B4] Álvarez-Martínez F. J. Barrajón-Catalán E. Micol V. (2020). Tackling antibiotic resistance with compounds of natural origin: a comprehensive review. Biomedicines 8, 405. doi: 10.3390/biomedicines8100405 33050619 PMC7601869

[B5] Beccaccioli M. Pucci N. Salustri M. Scortichini M. Zaccaria M. Momeni B. . (2022). Fungal and bacterial oxylipins are signals for intra- and inter-cellular communication within plant disease. Front. Plant Sci. 13. doi: 10.3389/fpls.2022.823233 36186042 PMC9524268

[B6] Boyce K. McLauchlan A. Schreider L. Andrianopoulos A. (2015). Intracellular growth is dependent on tyrosine catabolism in the dimorphic fungal pathogen Penicillium marneffei. PloS Pathog. 11, e1004790. doi: 10.1371/journal.ppat.1004790 25812137 PMC4374905

[B7] Bradford M. M. (1976). A rapid and sensitive method for the quantitation of microgram quantities of protein utilizing the principle of protein-dye binding. Anal. Biochem. 72, 248–254. doi: 10.1016/0003-2697(76)90527-3 942051

[B8] Burt S. (2004). Essential oils: their antibacterial properties and potential applications in foods—a review. Int. J. Food Microbiol. 94, 223–253. doi: 10.1016/j.ijfoodmicro.2004.03.022 15246235

[B9] Chen C. Z. Cooper S. L. (2002). Interactions between dendrimer biocides and bacterial membranes. Biomaterials 23, 3359–3368. doi: 10.1016/s0142-9612(02)00036-4 12099278

[B10] Cheng Y. J. Wu Y. J. Lee F. W. Ou L. Y. Chen C. N. Chu Y. Y. . (2022). Impact of storage condition on chemical composition and antifungal activity of pomelo extract against Colletotrichum gloeosporioides and anthracnose in post-harvest mango. Plants (Basel) 11 (15), 2064. doi: 10.3390/plants11152064 35956542 PMC9370353

[B11] Cruz A. G. Díaz-Jiménez L. Enríquez-Vara J. Mtz-Enríquez A. I. Quiñones-Aguilar E. E. Ramos-González R. . (2024). Control of phytopathogen organisms using bioactive compounds contained in orange wax. Biofuels Bioprod. Biorefin. 18, 510–523. doi: 10.1002/bbb.2582 41531421

[B12] David T. Mennat E. G. Federico R. Antonio D. P. (2015). Fungal pathogen uses sex pheromone receptor for chemotropic sensing of host plant signals. Nature 527, 521–524. doi: 10.1038/nature15516 26503056

[B13] Ene I. V. Brunke S. Brown A. J. P. Hube B. (2014). Metabolism in fungal pathogenesis. Cold Spring Harbor Perspect. Med. 4, a019695. doi: 10.1101/cshperspect.a019695 25190251 PMC4292087

[B14] Gai X. T. Jiang N. Lu C. H. Xia Z. Y. He Y. S. (2021). First report of tobacco fusarium root rot caused by Fusarium verticillioides in China. Plant Dis. 105, 3762. doi: 10.1094/pdis-03-21-0675-pdn

[B15] Gai X. Li S. Jiang N. Sun Q. Xuan Y. H. Xia Z. (2022). Comparative transcriptome analysis reveals that ATP synthases regulate Fusarium oxysporum virulence by modulating sugar transporter gene expressions in tobacco. Front. Plant Sci. 13, 978951. doi: 10.3389/fpls.2022.978951 36061782 PMC9433920

[B16] Han Y. Sun Z. Chen W. (2019). Antimicrobial susceptibility and antibacterial mechanism of limonene against Listeria monocytogenes. Molecules 25 (1), 33. doi: 10.3390/molecules25010033 31861877 PMC6982812

[B17] He Y. Han Q. Ayaz M. Chi Y. Xu A. Wang T. . (2025). Identification, characterization, and novel combination of fungicides against Fusarium pseudograminearum causing wheat crown rot in Anhui Province, China. Plant Dis. 109, 1922–1929. doi: 10.1094/pdis-01-25-0182-re 40965432

[B18] Ibáñez M. D. Sanchez-Ballester N. M. Blázquez M. A. (2020). Encapsulated limonene: a pleasant lemon-like aroma with promising application in the agri-food industry. a review. Molecules 25, 2598. doi: 10.3390/molecules25112598 32503168 PMC7321087

[B19] Jian Y. Chen X. Ma H. Zhang C. Luo Y. Jiang J. . (2023). Limonene formulation exhibited potential application in the control of mycelial growth and deoxynivalenol production in Fusarium graminearum. Front. Microbiol. 14, 1161244. doi: 10.3389/fmicb.2023.1161244. Epub 20230323. 37125209 PMC10131186

[B20] Karalija E. Macanović A. Ibragić S. (2025). Revisiting traditional medicinal plants: integrating multiomics, *in vitro* culture, and elicitation to unlock bioactive potential. Plants 14, 2029. doi: 10.3390/plants14132029 40648038 PMC12252194

[B21] Ke H. Rongyu L. Feixu M. Yi D. Aiai Z. Xue G. . (2023). Natural product osthole can significantly disrupt cell wall integrity and dynamic balance of Fusarium oxysporum. Pestic. Biochem. Physiol. 196, 105623–105623. doi: 10.1016/j.pestbp.2023.105623 37945232

[B22] Kim H. Kim M. H. Choi U. L. Chung M. S. Yun C. H. Shim Y. . (2024). Molecular and phenotypic investigation on antibacterial activities of limonene isomers and its oxidation derivative against Xanthomonas oryzae pv. oryzae. J. Microbiol. Biotechnol. 34, 562–569. doi: 10.4014/jmb.2311.11016 38247219 PMC11016764

[B23] Kimotho R. N. Maina S. (2024). Unraveling plant–microbe interactions: can integrated omics approaches offer concrete answers? J. Exp. Bot. 75, 1289–1313. doi: 10.1093/jxb/erad448 37950741 PMC10901211

[B24] Krishnakumar S. V. Mohammed Syed A. R. P I. V. Perumal R. Sampathrajan V. K. Nallusamy S. . (2025). Exploring the antifungal potential of citrus essential oils from Northeast India: a comparative gas chromatography–mass spectrometry, *in vitro*, and in silico analysis. ACS Agric. Sci. Technol. 5, 763–776. doi: 10.1021/acsagscitech.4c00665

[B25] Lin H. Li Z. Sun Y. Zhang Y. Wang S. Zhang Q. . (2024). D-limonene: promising and sustainable natural bioactive compound. Appl. Sci. 14 (11), 4605. doi: 10.3390/app14114605 30654563

[B26] Liu H. Zhang Y. Li H. Chen S. Zhang J. Ding W. (2024). Characteristics of soil microbial community assembly patterns in fields with serious occurrence of tobacco fusarium wilt disease. Front. Microbiol. 15. doi: 10.3389/fmicb.2024.1482952 39606108 PMC11600729

[B27] Makambi W. Ikonomova S. Karlsson A. (2023). Quantifying the antifungal activity of peptides against Candida albicans. J. Visualized Exp. 191, 64416. doi: 10.3791/64416 PMC10115183 36715417

[B28] Marei G. I. K. Rasoul M. A. A. Abdelgaleil S. A. M. (2012). Comparative antifungal activities and biochemical effects of monoterpenes on plant pathogenic fungi. Pestic. Biochem. Physiol. 103, 56–61. doi: 10.1016/j.pestbp.2012.03.004 38826717

[B29] Matelionienė N. Žvirdauskienė R. Kadžienė G. Zavtrikovienė E. Supronienė S. (2024). *In vitro* sensitivity test of Fusarium species from weeds and non-gramineous plants to triazole fungicides. Pathogens 13, 160. doi: 10.3390/pathogens13020160 38392898 PMC10892909

[B30] Matilla M. Krell T. (2017). The effect of bacterial chemotaxis on host infection and pathogenicity. FEMS Microbiol. Rev. 42 (1), fux052. doi: 10.1093/femsre/fux052 29069367

[B31] Michielse C. B. Rep M. (2009). Pathogen profile update: Fusarium oxysporum. Mol. Plant Pathol. 10, 311–324. doi: 10.1111/j.1364-3703.2009.00538.x 19400835 PMC6640313

[B32] Mihajlovic M. Rekanovic E. Hrustic J. Grahovac M. Pešić B. (2021). *In vitro* and *in vivo* toxicity of fungicides and biofungicides for the control of verticillium and fusarium wilt of pepper. Pesticidi i fitomedicina 36, 23–34. doi: 10.2298/pif2101023m

[B33] Mondal P. C. Kaushik P. Pankaj Shakil N. A. Rana V. S. (2026). Chemical composition, nematicidal and fungicidal activities of the essential oil of Mentha spicata against Meloidogyne incognita and Fusarium oxysporum f.sp. lycopersici. Chem. Biodivers. 23, e02351. doi: 10.1002/cbdv.202502351 41317391

[B34] Morcia C. Tumino G. Ghizzoni R. Bara A. Salhi N. Terzi V. (2017). *In vitro* evaluation of sub-lethal concentrations of plant-derived antifungal compounds on FUSARIA growth and mycotoxin production. Molecules 22, 1271. doi: 10.3390/molecules22081271 28758914 PMC6151992

[B35] Muñoz Castellanos L. Amaya Olivas N. Ayala-Soto J. De La O Contreras C. M. Zermeño Ortega M. Sandoval Salas F. . (2020). *In Vitro* and *In Vivo* Antifungal Activity of Clove (Eugenia caryophyllata) and Pepper (Piper nigrum L.) Essential Oils and Functional Extracts Against Fusarium oxysporum and Aspergillus niger in Tomato (Solanum lycopersicum L.). Int J Microbiol. 2020, 1702037. doi: 10.1155/2020/1702037 32399036 PMC7211242

[B36] Pandey R. (2023). Controlling tobacco diseases: an overview of black shank and fusarium wilt. Int. J. Appl. Sci. Biotechnol. 11, 1–7. doi: 10.3126/ijasbt.v11i1.52440 37609976

[B37] Porras G. Chassagne F. Lyles J. T. Marquez L. Dettweiler M. Salam A. M. . (2021). Ethnobotany and the role of plant natural products in antibiotic drug discovery. Chem. Rev. 121, 3495–3560. doi: 10.1021/acs.chemrev.0c00922 33164487 PMC8183567

[B38] Qiu R. Li X. Li C. Li C. Xue C. Fang W. . (2022). First report of tobacco root rot caused by Fusarium falciforme in China. Plant Dis. 107 (3). doi: 10.1094/pdis-06-22-1394-pdn

[B39] Santacruz D. Enane F. O. Fundel-Clemens K. Giner M. Wolf G. Onstein S. . (2022). Automation of high-throughput mRNA-seq library preparation: a robust, hands-free and time efficient methodology. SLAS Discov. 27, 140–147. doi: 10.1016/j.slasd.2022.01.002 35093290

[B40] Shen H. F. Yang Q. Y. Pu X. M. Zhang J. X. Sun D. Y. Liu P. P. . (2023). First report of fusarium root rot of tobacco caused by Fusarium fujikuroi in Guangdong Province, China. Plant Dis. 107, 3294. doi: 10.1094/pdis-03-26-0477-pdn

[B41] Veloso J. Díaz J. (2021). The non-pathogenic Fusarium oxysporum Fo47 induces distinct responses in two closely related solanaceae plants against the pathogen Verticillium dahliae. J. Fungi 7, 344. doi: 10.3390/jof7050344 33925134 PMC8146752

[B42] Víchová J. Jílková B. Michutová M. Kmoch M. (2024). *In vitro* and *in vivo* antibacterial activity of selected essential oil components against Pectobacterium carotovorum subsp. carotovorum and Pectobacterium atrosepticum causing bacterial soft rot of potato tubers. Heliyon 10, e32081. doi: 10.1016/j.heliyon.2024.e32081 38882333 PMC11177147

[B43] Viñas M. Karlovsky P. (2025). A comprehensive review of hypotheses about the biological function of zearalenone, and a new hypothesis for the function of resorcylic and dihydroxyphenylacetic macrolactones in fungi. Toxins (Basel) 17 (5), 226. doi: 10.3390/toxins17050226 40423309 PMC12115441

[B44] Wang L. Qiu M. Li X. Liu M. Li L. Chen Y. (2025). Antimicrobial activity and possible mechanisms of juglone against Escherichia coli, Staphylococcus aureus, and Salmonella pullorum. BMC Microbiol. 25, 668. doi: 10.1186/s12866-025-04354-0 41107751 PMC12534947

[B45] Xia Q. Dong P. (2025). Antifungal activity and mechanism of limonene against Fusarium oxysporum, a pathogen of potato dry rot. bioRxiv. doi: 10.1101/2025.10.06.675995

[B46] Yang Y. Li Y. Mei X. Yang M. Huang H. Du F. . (2022). Antimicrobial terpenes suppressed the infection process of Phytophthora in fennel-pepper intercropping system. Front. Plant Sci. 13, 890534. doi: 10.3389/fpls.2022.890534. Epub 20220609. 35755704 PMC9218821

[B47] Zhang X. Hu X. Sun Z. Wang J. Xu H. Tian Y. (2025). Control of tobacco Fusarium root rot by Bacillus amyloliquefaciens HN11 and its influence on the rhizosphere microbial community. Pest Manage. Sci. 82, 3532–3543. doi: 10.1002/ps.70474 41454425

[B48] Zhang X. Peng W. Zheng M. Yang B. Tai B. Li X. . (2024). Data-independent acquisition proteome technology for analysis of antifungal and anti-aflatoxigenic properties of eugenol to aspergillus flavus. J. Agric. Food Chem. doi: 10.1021/acs.jafc.4c04635 39352270

[B49] Zhou L.-R. Hu H.-J. Wang J. Zhu Y.-X. Zhu X.-D. Ma J.-W. . (2024). Cinnamaldehyde acts as a fungistat by disrupting the integrity of Fusarium oxysporum Fox-1 cell membranes. Horticulturae 10, 48. doi: 10.3390/horticulturae10010048 30654563

[B50] Zhou S. W. Zhu Y. Qin X. J. Li Y. Noiprasert S. Apiwongsrichai K. . (2025). D-limonene inhibits the growth of Fusarium proliferatum by decreasing H3K9ac and H3K27ac modifications. BMC Genomics 27, 55. doi: 10.1186/s12864-025-12389-w 41388511 PMC12817427

[B51] Zhu Y. Li M. Li K. Li J. Liu X. Yang S. . (2024). Integrated metabolome and transcriptome analyses reveal the critical role of alpha-linolenic acid metabolism in Panax notoginseng root rot disease. Ind. Crops Prod. 222, 120072. doi: 10.1016/j.indcrop.2024.120072 38826717

